# Human-in-the-Loop Performance of LLM-Assisted Arterial Blood Gas Interpretation: A Single-Center Retrospective Study

**DOI:** 10.3390/jcm14186676

**Published:** 2025-09-22

**Authors:** Sergio Ayala-De la Cruz, Paola Elizabeth Arenas-Hernández, María Fernanda Fernández-Herrera, Rebeca Alejandrina Quiñones-Díaz, Jorge Martín Llaca-Díaz, Erik Alejandro Díaz-Chuc, Diana Guadalupe Robles-Espino, Erik Alejandro San Miguel-Garay

**Affiliations:** 1 Facultad de Medicina y Hospital Universitario “Dr. José Eleuterio González”, Departamento de Patología Clínica, Universidad Autónoma de Nuevo León, Monterrey 64460, Nuevo León, Mexico; sergio.ayalad@uanl.edu.mx (S.A.-D.l.C.); jorge.llacadz@uanl.edu.mx (J.M.L.-D.); ediaz.me0085@uanl.edu.mx (E.A.D.-C.); drobles.084470@uanl.edu.mx (D.G.R.-E.); 2 Facultad de Medicina y Hospital Universitario “Dr. José Eleuterio González”, Universidad Autónoma de Nuevo León, Monterrey 64460, Nuevo León, Mexico; paola.arenashrnd@uanl.edu.mx (P.E.A.-H.); maria.fernandezha@uanl.edu.mx (M.F.F.-H.); rebeca.quinonesdz@uanl.edu.mx (R.A.Q.-D.)

**Keywords:** generative artificial intelligence, blood gas analysis, acid–base imbalance, medical students

## Abstract

**Background and Objectives**: Interpreting acid–base disorders is challenging, particularly in complex or mixed cases. Given the growing potential of large language models (LLMs) to assist in cognitively demanding tasks, this study evaluated their performance in interpreting arterial blood gas (ABG) results. **Materials and Methods**: In this single-center retrospective study, 200 ABG datasets were curated to include 40 cases in each of five diagnostic categories: metabolic acidosis, respiratory acidosis, metabolic alkalosis, respiratory alkalosis, and no acid–base disorder. Three medical students, each assigned to one LLM (ChatGPT GPT-4o, Copilot GPT-4, or Gemini 1.5-flash/2.5-flash), perform ABG interpretation using two evaluation methods: interpretation (LLM-I) and interpretation with supervision model (LLM-S). Two clinical pathologists independently performed the conventional evaluation to serve as the reference standard. **Results**: Agreement for identifying the primary acid–base (APD) disorder was strong across all approaches (Cohen’s κ ≥ 0.88). For identifying both primary and secondary disorders regardless of order (APSD), LLM-I showed moderate agreement (ChatGPT κ = 0.65, Copilot κ = 0.61, Gemini κ = 0.62), whereas LLM-S achieved strong agreement (ChatGPT κ = 0.91, Copilot κ = 0.81, Gemini κ = 0.81). **Conclusions**: LLM-assisted ABG interpretation demonstrates strong concordance with expert interpretation in detecting primary acid–base disorders. These tools may enhance the understanding of acid–base disorders while reducing calculation-related errors among medical students.

## 1. Introduction

Large language models (LLMs) are artificial intelligence systems based on transformer architectures, trained on massive volumes of data to generate natural text that emulates human writing. Among these models, ChatGPT (Chat Generative Pre-Trained Transformer) has gained the widest recognition. Its applications span text summarization, translation, code generation, and answering complex questions, among many others [1,2].

In medicine, LLMs have demonstrated strong performance in medical licensing examinations [3]. However, their role in clinical diagnosis and decision-making remains under investigation. For example, Hager et al. (2024) found that while LLMs achieved high diagnostic accuracy in abdominal pathologies, their performance was still inferior to that of physicians [3]. Nevertheless, LLMs hold promise in laboratory medicine, both as educational tools and as aids in the interpretation of clinical studies [4,5]. Trust in AI is growing: 58% of medical students believe that AI-based evaluations are more objective than traditional methods [6], and 90% expect their routine integration within the next decade [7]. Still, before broad clinical adoption, LLMs must undergo rigorous validation with specialized medical knowledge.

Acid–base disorders are common in critically ill patients, and their evaluation remains challenging, as it requires identification of both the primary disturbance and the compensatory response, particularly in mixed disorders [8,9]. The physiological evaluation approach involves:

1. Determining acidosis or alkalosis via pH measurement.
2. Identifying the primary disorder (metabolic or respiratory) based on changes in bicarbonate (HCO_3_^−^) and arterial partial pressure of carbon dioxide (PaCO_2_), respectively.
3. Assessing compensatory mechanisms, which typically involve respiratory response to metabolic disorders and vice versa.
4. In metabolic acidosis, calculating the plasma anion gap to refine etiological assessment.

The term “compensation” describes the secondary response, as it originates from the physiological buffering mechanisms [10]. While HCO_3_^−^ and PaCO_2_ usually change in the same direction, deviations in magnitude or direction may indicate additional disorders [8]. However, evaluation is complicated by the need for multiple empirically derived compensation formulas, susceptibility to arithmetic errors (e.g., error on signs, decimal and parentheses placements), and subjective interpretation. These challenges suggest a potential role for LLMs in acid–base interpretation, as they combine computational accuracy with explanatory capacity [4].

The aim of this study was to assess the performance of three LLMs in assisting the interpretation of arterial blood gases (ABG), with a focus on their diagnostic accuracy for acid–base disorders.

## 2. Materials and Methods

### 2.1. Study Design and Patient Population

This retrospective observational study was conducted between December 2024 and June 2025 at the “Dr. José E. González” University Hospital, affiliated with the Universidad Autónoma de Nuevo León. The protocol was approved by the institutional ethics committee (Protocol No. PC24-00003).

The dataset included the first available ABG measurement from adult patients (≥18 years) attended between January 2023 and November 2024. Exclusion criteria were hypoalbuminemia (serum albumin < 4.0 g/dL; the rationale for this exclusion is provided in Section 2.2), missing serum sodium or chloride values, a time gap > 2 h between ABG and serum electrolyte sampling, and insufficient data to classify respiratory disorders as acute (<4 days) or chronic (≥4 days) [8,9]. For patients without a respiratory disorder, chronicity status was randomly assigned.

Sample size was calculated using the method recommended by Donner and Rotondi (2010) [11] for agreement studies. The *kappaSize* package in R was employed, applying the *CI5Cats* function for five diagnostic categories (four primary acid–base disorders and one category with no disorder). Parameters included equal category proportions (0.20), κ_low_ = 0.60, κ_estimate_ = 0.67, α = 0.05, and the null hypothesis of weak or worse agreement (κ < 0.60) [11,12]. The minimum required sample size was 188; therefore, 200 cases were selected (40 per category). Eligible ABG cases were reviewed consecutively and in chronological order by one of the evaluators from the conventional evaluation team, and cases were included until the quota of 40 per category was reached. Reference ranges were pH 7.35–7.45, PaCO_2_ 35–45 mmHg, HCO_3_^−^ 22–26 mmol/L, sodium 135–145 mmol/L, and chloride 99–110 mmol/L. Temperature, FiO_2_ and altitude were not inputs.

### 2.2. Conventional Evaluation

Two board-certified clinical pathologists performed the conventional evaluation blinded to each other and to LLM outputs. In cases of disagreement, a third pathologist was to review the case, and the final classification was determined based on concordance between any two of the three evaluators. Both evaluators were aware of the study design in advance, including the interpretation criteria, cutoffs, and equal category sampling. Compensation calculations were generated using software to avoid arithmetic errors.

Acid–base status was assessed using the physiological method, which involved: (1) verifying internal consistency with the Henderson–Hasselbalch equation; (2) determining acidemia or alkalemia; (3) classifying the primary disorder as metabolic or respiratory; (4) assessing compensation for the primary disorder; (5) calculating of the anion gap (AG); and (6) calculating of the delta/delta (Δ/Δ) ratio when AG was elevated [8,13,14].

Equations (1)–(8) were used for compensation, AG, and Δ/Δ ratio calculations [8,13,15]. Berend et al. (2014) [8] recommend a tolerance of ±2 units for expected PaCO_2_ in metabolic acidosis and metabolic alkalosis to define an appropriate compensatory response. Rodríguez-Villar et al. (2020) [13] recommend a tolerance of ±2 units for both expected PaCO_2_ and HCO_3_^−^ across all disorders to establish appropriate compensation. Accordingly, a tolerance of ±2 units for both expected PaCO_2_ and HCO_3_^−^ was adopted in this study. An AG >12 mmol/L was considered elevated [13,15]. For the Δ/Δ ratio (only for metabolic acidosis with AG elevated), values between 1–2 indicated isolated high-AG metabolic acidosis, <1 suggested concurrent high-AG and non-AG metabolic acidosis, and >2 suggested high-AG metabolic acidosis with concomitant chronic respiratory acidosis or metabolic alkalosis [13,14,16].

Metabolic acidosis [8]:

Expected PaCO_2_ = (1.5 × HCO_3_^−^) + 8 ± 2 (1)

Acute respiratory acidosis [8]:

Expected HCO_3_^−^ = 24 + 0.1 × (PaCO_2_ − 40) ± 2 (2)

Chronic respiratory acidosis [8]:

Expected HCO_3_^−^ = 24 + 0.4 × (PaCO_2_ − 40) ± 2 (3)

Metabolic alkalosis [8]:

Expected PaCO_2_ = 40 + 0.7 × (HCO_3_^−^ − 24) ± 2 (4)

Acute respiratory alkalosis [8]:

Expected HCO_3_^−^ = 24 − 0.2 × (40 − PaCO_2_) ± 2 (5)

Chronic respiratory alkalosis [8]:

Expected HCO_3_^−^ = 24 − 0.4 × (40 − PaCO_2_) ± 2 (6)

Anion gap (AG) [8]:

[Na^+^] − [Cl^−^] − [HCO_3_^−^] (7)

Delta/delta (Δ/Δ) [15]:

(AG − 12)/(24 − [HCO_3_^−^]) (8)

### 2.3. LLM-Assisted Evaluation

Three medical students currently completing their clinical internship, each assigned to one LLM (ChatGPT GPT-4o, OpenAI, San Francisco, CA, USA; Copilot GPT-4, Microsoft Corporation, Redmond, WA, USA; or Gemini 1.5-flash and 2.5-flash, Google LLC, Mountain View, CA, USA) performed ABG interpretation using two predefined LLM-assisted evaluation methods: LLM-assisted evaluation performed with interpretation (LLM-I) and performed with supervision (LLM-S).

**Figure 1:**
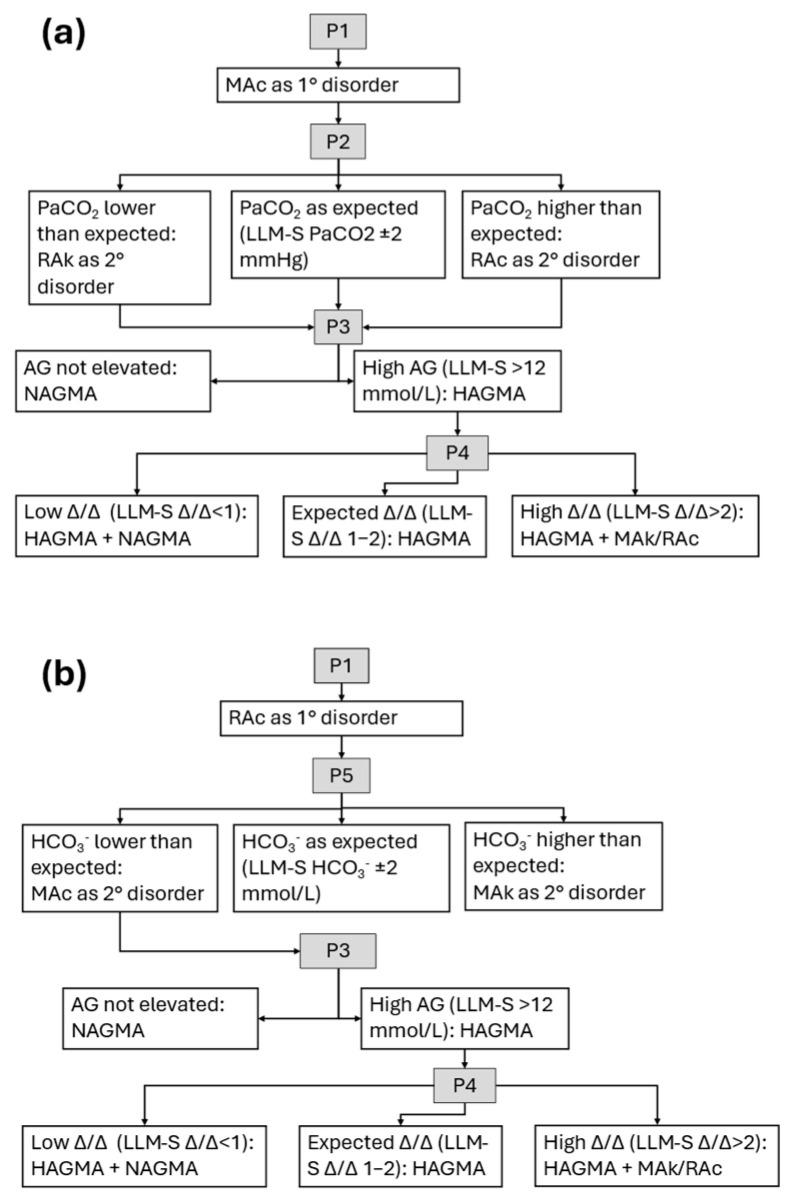
LLM-assisted evaluation of acidosis. Literature-based algorithms [8,9,13] for prompts (P1, P2, P3, etc.) are shown: (**a**) metabolic acidosis; (**b**) respiratory acidosis. AG, anion gap; HAGMA, high anion gap metabolic acidosis; NAGMA, normal anion gap metabolic acidosis; MAc, metabolic acidosis; MAk, metabolic alkalosis; P(number), prompt; RAc, respiratory acidosis; RAk, respiratory alkalosis. Δ/Δ, delta/delta ratio. LLM-S, LLM-assisted evaluation with supervision. All prompts are detailed in Table 1.

**Figure 2:**
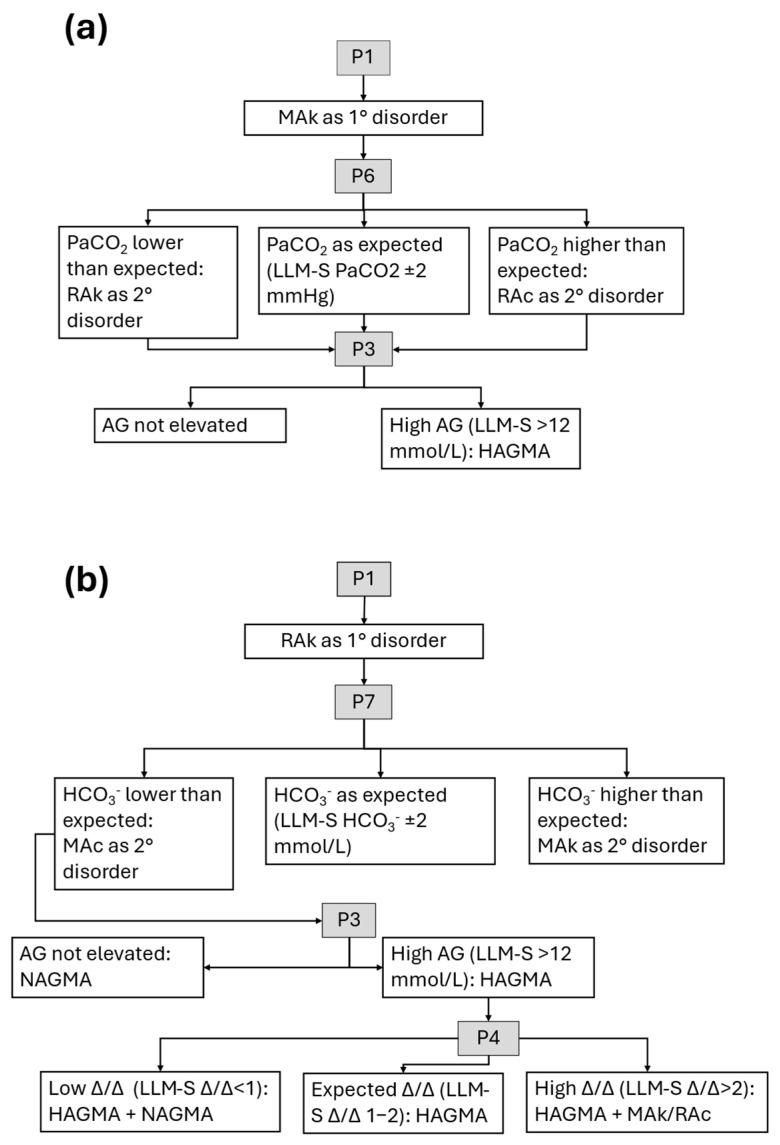
LLM-assisted evaluation of alkalosis. Literature-based algorithms [8,9,13] for prompts (P1, P2, P3, etc.) are shown: (**a**) metabolic alkalosis; (**b**) respiratory alkalosis. AG, anion gap; HAGMA, high anion gap metabolic acidosis; NAGMA, normal anion gap metabolic acidosis; MAc, metabolic acidosis; MAk, metabolic alkalosis; P(number), prompt; RAc, respiratory acidosis; RAk, respiratory alkalosis. Δ/Δ, delta/delta ratio. LLM-S, LLM-Assisted evaluation with supervision. All prompts are detailed in Table 1.

To standardize outputs, all initial prompts were pre-formulated in Spanish and generated via spreadsheet to avoid transcription errors. The aim of standardizing and adopting a literature-based algorithmic approach [8,9,13] was to minimize variability in the responses generated by the different LLMs (Figure 1 and Figure 2). The initial prompt, translated to English, followed this format:

*“Identify whether or not there is an acid-base disorder based on the following results. Reference values are shown in parentheses. pH = XX (7.35–7.45), PaCO_2_ = XX mmHg (35–45 mmHg), HCO_3_^−^ = XX mmol/L (22–26 mmol/L). The serum electrolyte results are: sodium = XX mmol/L, chloride = XX mmol/L. If an acid-base disorder is identified, determine the primary disorder.”*

**Table 1:** Prompts used in LLM-assisted evaluation.

Prompt	LLM-I	LLM-S
P2	Calculate the expected PaCO_2_ compensation in metabolic acidosis and determine whether an additional respiratory disorder is present.	Calculate the expected PaCO_2_ compensation in metabolic acidosis.
P3	Calculate and interpret the anion gap value.	Calculate the anion gap value.
P4	Calculate and interpret the delta/delta ratio using the changes in anion gap and HCO_3_^−^ values.	Calculate the delta/delta ratio using the changes in anion gap and HCO_3_^−^ values.
P5	Calculate the expected HCO_3_^−^ compensation in acute/chronic respiratory acidosis and determine whether an additional metabolic disorder is present.	Calculate the expected HCO_3_^−^ compensation in acute/chronic respiratory acidosis.
P6	Calculate the expected PaCO_2_ compensation in metabolic alkalosis and determine whether an additional respiratory disorder is present.	Calculate the expected PaCO_2_ compensation in metabolic alkalosis.
P7	Calculate the expected HCO_3_^−^ compensation in acute/chronic respiratory alkalosis and determine whether an additional metabolic disorder is present.	Calculate the expected HCO_3_^−^ compensation in acute/chronic respiratory alkalosis.

LLM-I, LLM-assisted evaluation with interpretation; LLM-S, LLM-assisted evaluation with supervision. Prompts were translated from Spanish.

Basic paid subscriptions (≈USD $20/month each) were used to avoid model downgrades and ensure access to the latest versions. To prevent potential influence from prior interactions, a new conversation was initiated for each case. The prompts were optimized through pilot testing to minimize response bias; for example, in cases without an acid–base disorder, we observed a tendency to force the detection of an acid–base disorder when the option of no disorder was not explicitly included. We also noted that providing reference ranges for sodium and chloride sometimes led the models to overinterpret mild deviations, and therefore these values were not included in the prompt. Furthermore, in the pilot testing we found that including cases with hypoalbuminemia prompted the LLM to attribute the finding to chronic liver disease and suggest the presence of respiratory alkalosis, even when the remaining data did not support such disorder. Notably, no issues were observed in the calculation of the albumin-corrected anion gap. To avoid this source of bias, cases with hypoalbuminemia were excluded.

#### 2.3.1. LLM-Assisted Evaluation with Interpretation (LLM-I)

In this method, conditional prompts were applied based on the outcome of the initial prompt. The model was instructed both to perform the calculations and to provide an interpretation of the results (Table 1, Figure 1 and Figure 2). When a respiratory disorder was identified, the chronicity data provided were used; however, to avoid bias, LLM users were informed that this variable was included in all cases (even when no respiratory disorder was present) and instructed to consider chronicity only when a respiratory disorder was detected.

#### 2.3.2. LLM-Assisted Evaluation with Supervision (LLM-S)

As in the LLM-I method, conditional prompts were used depending on the result of the initial prompt. The difference in this method was that the LLM was instructed to perform the calculations, but the interpretation is left to the user (Table 1, Figure 1 and Figure 2).

For the LLM-S method, the internal consistency of the calculations was assessed in relation to the expected direction of compensation. For example, in a primary metabolic acidosis, a decrease in PaCO_2_ is expected; it would be inconsistent for the LLM to calculate an increase in PaCO_2_ as a compensatory response. In such cases, an additional prompt specifying the expected direction of compensation was provided.

### 2.4. Statistical Analysis

Numerical variables are reported as median and interquartile range (IQR), categorical variables as percentages. Agreement between conventional and LLM-assisted evaluations was evaluated using Cohen’s kappa (κ) coefficient. Four agreement metrics were calculated:

- Agreement on primary disorder (APD): concordance between the LLM-assisted and the conventional method in identifying the primary acid–base disorder.
- Agreement on primary disorder with detection (APD-a): same as APD, but the LLM identified the primary disorder even if it appeared as a secondary disorder.
- Agreement on both primary and secondary disorders regardless of order (APSD): concordance between the two methods in identifying the same two disorders (primary and secondary), regardless of order.
- Agreement on the classification of metabolic acidosis (AMA): concordance between the two methods in classifying metabolic acidosis based on the AG.

Since Cohen’s κ may exhibit undesirable behavior in imbalanced datasets (i.e., a worse classifier gets higher κ) [17], a post hoc analysis was performed using a metric with better performance under such conditions, namely the *R_k_* coefficient (a generalization of the Matthews correlation coefficient for the multiclass case) [17,18]. Macro-average and micro-average metrics were computed for sensitivity, specificity, positive predictive value, negative predictive value, and F1, which are generalizations applicable for evaluating the performance of multiclass classifiers [19]. In addition, a sensitivity analysis was conducted for the tolerances applied in the compensation equations. For Equation (1) (Winters equation for metabolic acidosis), a tolerance of ±2 units was applied, as this is the value consistently reported in the literature [8,13,14,15]. For the remaining disorders, a tolerance of ±5 units was applied, as recommended by several authors for metabolic alkalosis and respiratory disorders [10,20].

Agreement was interpreted according to the κ value, as recommended by McHugh [12]: 0–0.20, none; 0.21–0.39, minimal; 0.40–0.59, weak; 0.60–0.79, moderate; 0.80–0.90, strong; and >0.90, almost perfect. Statistical analysis was conducted in R (version 4.3.1; R Foundation for Statistical Computing, Vienna, Austria) and RStudio (version 2023.9.1.494; Posit Software, Boston, MA, USA) using the “base,” “tidyverse,” “irr,” “mltools,” and “caret” packages.

## 3. Results

A total of 2288 patients with an ABG event were identified. From these, 200 cases were selected to represent primary five diagnostic categories: metabolic acidosis (MAc), respiratory acidosis (RAc), metabolic alkalosis (MAk), and respiratory alkalosis (RAk), and no acid–base disorder (NoABD), with 40 cases per category. For analysis purposes, the absence of both primary and secondary disorders was treated as a distinct category. The agreement between the two evaluators in the conventional evaluation was as follows: APD, κ = 1.0 (95% CI: 0.93–1.0); APSD, κ = 1.0 (95% CI: 0.94–1.0); and AMA, κ = 1.0 (95% CI: 0.90–1.0).

The median patient age was 50 years (IQR 39–65 years), and 82 patients (41%) were female. Most cases (172; 86%) were from the emergency department, 15 (8%) from internal medicine and 13 (7%) from surgical services. The median interval between ABG and serum electrolyte sampling was 28.6 min (IQR 12.6–62.7 min). Table 2 summarizes demographic and ABG characteristics by primary disorder category. A total of 74 cases (37%) involved mixed disorders, distributed as follows (regardless of order): 19 (9.5%) MAc/RAc, 16 (8.0%) MAc/RAk, 6 (3.0%) RAc/MAk, and 33 (16.5%) MAk/RAk.

The LLM-I method was applied from 22 January to 28 February 2025, and the LLM-S method from 2 April to 20 June 2025, for Gemini and Copilot. ChatGPT LLM-S evaluation was conducted between 3 June and 20 June 2025.

For LLM-I, agreement with the conventional evaluation for identifying the primary disorder (APD) was κ = 0.91 for ChatGPT, κ = 0.95 for Copilot, and κ = 0.88 for Gemini (strong and almost perfect agreement). Agreement for detecting the primary disorder with detection (APD-a) was high across all models (κ ≥ 0.94). In contrast, agreement for identifying both primary and secondary disorders regardless of order (APSD) was lower, with κ values ranging from 0.61 to 0.65 (moderate agreement) (Table 3).

For the classification of metabolic acidosis by anion gap (AMA), 52 cases were included for ChatGPT and Gemini, and 51 cases for Copilot, regardless of whether it was deemed a primary or secondary disorder. κ values ranged from 0.48 to 0.76, with only Gemini showing moderate agreement (κ = 0.76).

In the LLM-S method, APSD agreement improved substantially, with κ = 0.91 for ChatGPT and κ = 0.81 for both Copilot and Gemini. AMA classification also improved, with κ ≥ 0.85 across all models (strong agreement) (Table 3). *R_k_* values were very similar to κ in most cases, and no instance was identified in which κ was inappropriately higher than *R_k_*.

**Figure 3:**
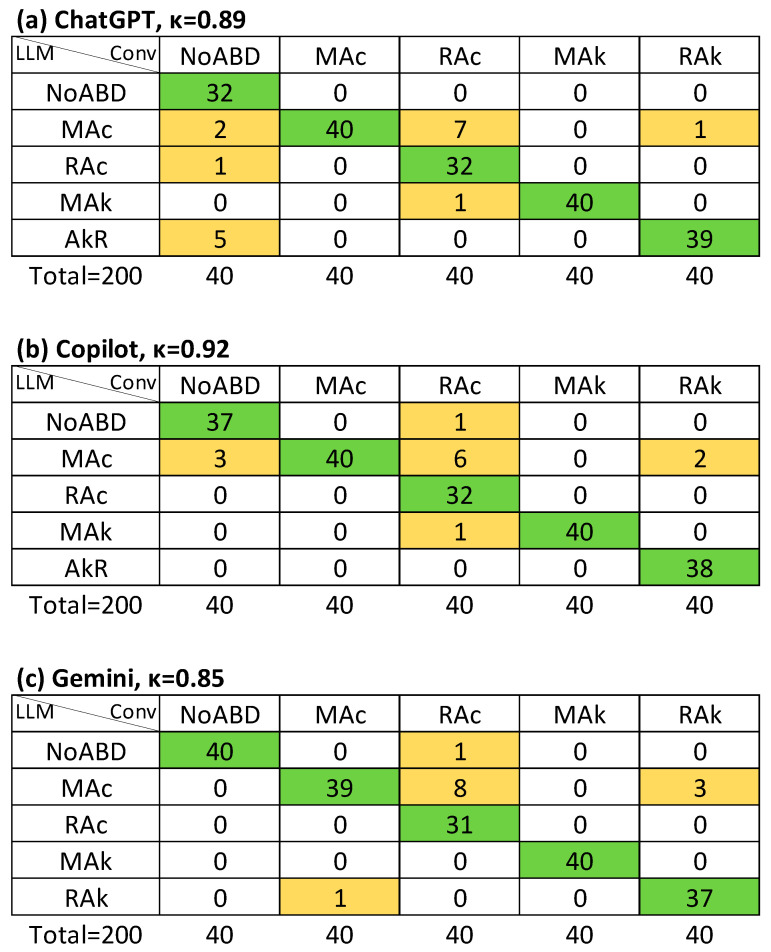
Agreement (Cohen’s κ value) on primary disorder between LLM-assisted with supervision and conventional method (APD): (**a**) ChatGPT; (**b**) Copilot; (**c**) Gemini. Conv, conventional evaluation (column titles), LLM, large language model assisted evaluation (row titles); NoABD, no acid–base disorder; MAc, metabolic acidosis; MAk, metabolic alkalosis; RAc, respiratory acidosis; RAk, respiratory alkalosis. Cells indicate agreement frequencies: green = concordant cases, orange = discordant cases.

**Figure 4:**
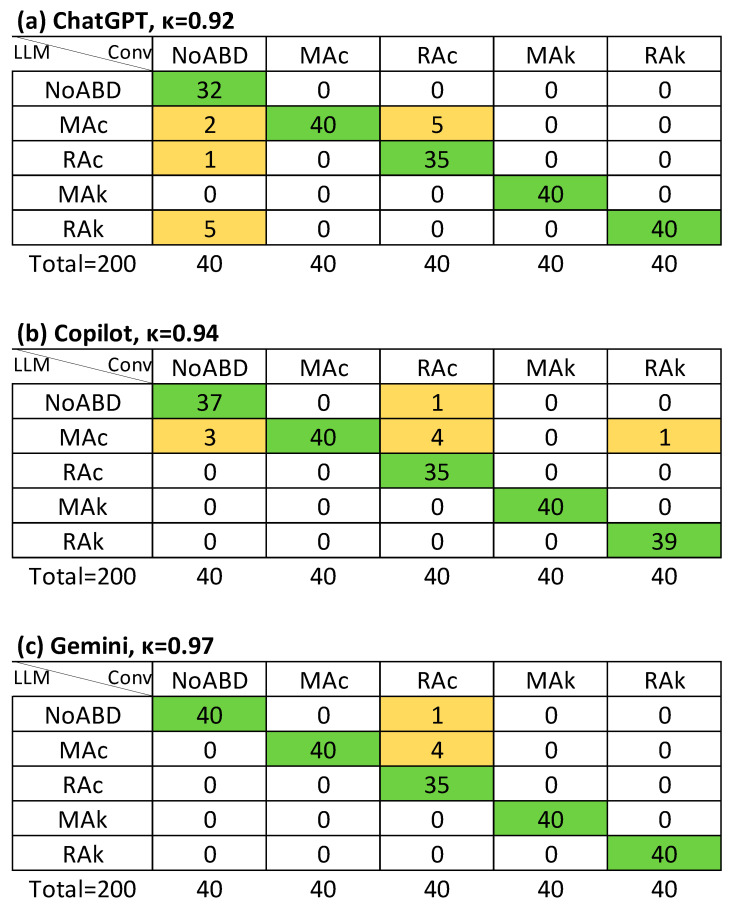
Agreement (Cohen’s κ value) on primary disorder between LLM-assisted with supervision and conventional method even if it appeared as a secondary disorder. (APD-a): (**a**) ChatGPT; (**b**) Copilot; (**c**) Gemini. Conv, conventional evaluation (column titles), LLM, large language model assisted evaluation (row titles). NoABD, no acid–base disorder. MAc, metabolic acidosis; MAk, metabolic alkalosis; RAc, respiratory acidosis; RAk, respiratory alkalosis. Cells indicate agreement frequencies: green = concordant cases, orange = discordant cases.

**Figure 5:**
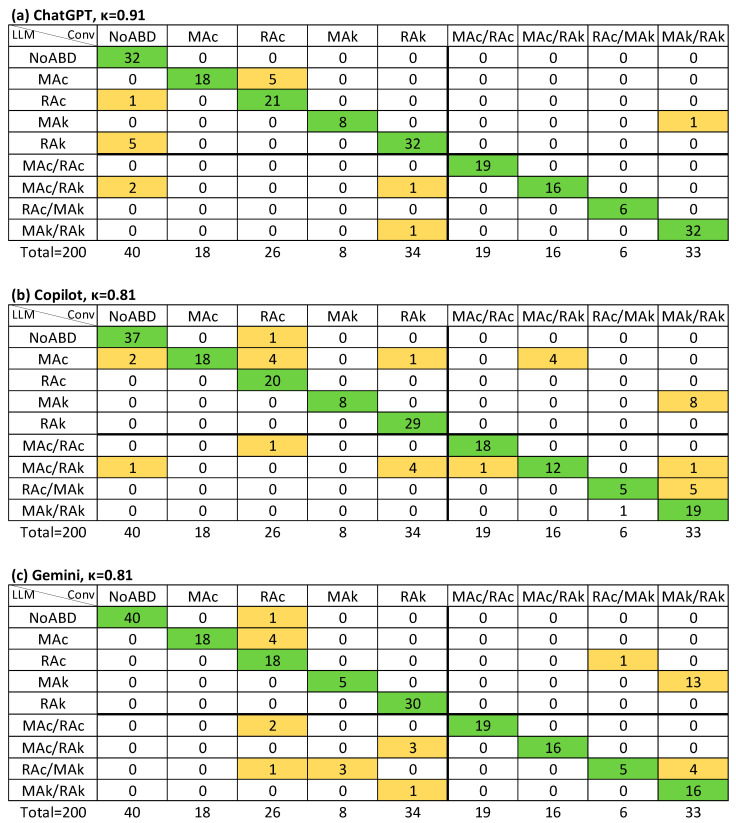
Agreement (Cohen’s κ value) on both primary and secondary disorders regardless of order between LLM-assisted with supervision and conventional method (APSD): (**a**) ChatGPT; (**b**) Copilot; (**c**) Gemini. Conv, conventional evaluation (column titles), LLM, large language model assisted evaluation (row titles); NoABD, no acid–base disorder; MAc, metabolic acidosis; MAk, metabolic alkalosis; RAc, respiratory acidosis; RAk, respiratory alkalosis. Cells indicate agreement frequencies: green = concordant cases, orange = discordant cases.

Figure 3, Figure 4 and Figure 5 illustrate the agreement levels for APD, APD-a, and APSD in the LLM-S evaluation, respectively. Errors in the LLM-S evaluations included a failure to impute a primary disorder in a mixed case for ChatGPT; two errors for Copilot (a misapplication of a chronic compensation formula to an acute metabolic alkalosis, and an incorrect directional change in bicarbonate for respiratory alkalosis due to an arithmetic sign error); and four failures to impute a primary disorder for Gemini (three mixed cases and one simple case).

Two sub-analyses were conducted to further assess LLM performance. In the first, cases were selected based on the presence of pronounced acidemia (pH ≤ 7.30) or alkalemia (pH ≥ 7.50), yielding 68 cases (34%). Overall, the results were consistent with those from the full dataset, and the LLM-S method again outperformed LLM-I (Table 4). The second sub-analysis, referred to as “severe secondary disorders”, included cases with an expected compensation exceeded 4 units (e.g., a metabolic acidosis with an expected PaCO_2_ of 30 mmHg but an observed PaCO_2_ > 34 mmHg), resulting in 31 cases. Notably, ChatGPT under the LLM-I method showed improved performance compared to the full-case analysis.

In the sensitivity analysis (SA), 33 of the 74 cases that had been classified as mixed disorders in the a priori analysis were reclassified as simple disorders (Supplementary Figure S1), and the agreement between both methods was strong (κ = 0.81, IC95% 0.76–0.86) in the APSD analysis. The agreement between LLM-I and SA was comparable to that observed with the a priori method, except for ChatGPT, where it decreased to κ = 0.51 in APSD (Supplementary Table S3), compared with κ = 0.65 in the a priori analysis. The macro-average values of the diagnostic performance metrics are shown in Table 5. Overall, good performance was observed in the APD and APD-a analyses for both LLM-I and LLM-S (macro-averaged sensitivity and specificity >0.90); however, in the APSD and AMA analyses, LLM-S demonstrated superior performance, particularly in the case of ChatGPT (macro-averaged sensitivity and specificity ≥0.95).

## 4. Discussion

This study demonstrates strong agreement between LLM-assisted ABG interpretation and conventional expert evaluation, particularly in detecting primary acid–base disorders.

Previous work has reported more variable performance. Lee et al. (2024) [21] compared interpretations by a nephrologist, ChatGPT, and a clinical calculator in 130 intensive care unit (ICU) patients, finding poor overall agreement (Fleiss’ κ = −0.14). Nephrologists concluded that just one (0.8%) had no acid–base disorder and four (3%) had an isolated primary disorder. In contrast, ChatGPT failed to detect any disorder in 21% of cases with at least one disorder and not identified mixed disorders in 41% of cases. In contrast, in our dataset (comprising mainly emergency department patients), missed diagnoses were rare. Only one case across both LLM-assisted evaluation methods was misclassified as “no disorder” despite an existing imbalance (Figure 4). 74 cases (37%) involved mixed disorders. Under the LLM-I method with ChatGPT, from the 74 cases with mixed disorders, five (7%) were misclassified as isolated primary disorders, whereas under LLM-S, only one (1.4%) was misclassified as it (Figure 5).

Several factors may explain this discrepancy. Case complexity differed: Lee et al.’s sample included only ICU patients and was dominated by mixed disorders, whereas our dataset included a more balanced representation of simple and mixed cases. The ChatGPT version may also have influenced results; our evaluations used GPT-4o [22] via a paid subscription, which likely outperforms older or free-tier versions that revert to a worse LLM version (GPT-3.5 during the study period) after exceeding usage limits. Lee et al. did not specify the version used, but we subjectively noted worse performance with GPT-3.5 during prompt design.

Gemini’s improved performance in LLM-S compared to LLM-I may partly reflect its model update from Gemini-1.5-flash to Gemini-2.5-flash during the study period (17 June 2025) [23]. However, this transition occurred only at the end of the LLM-S evaluation period, meaning that most of the cases were still assessed with Gemini-1.5-flash. In contrast, ChatGPT’s version remained stable across both methods, yet performance still improved under LLM-S, especially in mixed disorders classification. Copilot uses GPT-4, but the exact versions and release timelines are not publicly disclosed [24]. This suggests that supervision, and not just model updates, can enhance accuracy.

Our findings are consistent with Gün (2025) [25], who reported high agreement between LLMs and an emergency physician for isolated primary disorders. Agreement was ≥90% for pulmonary disorders, 80–90% for diabetic ketoacidosis, lactic acidosis, and acute kidney injury, but <70% for mixed and toxicological acid–base disorders. In our study, LLM-I achieved only weak-to-moderate agreement (κ = 0.61–0.65) for identifying both primary and secondary disorders, while LLM-S achieved strong agreement (κ = 0.81–0.91). Also, strong agreement for primary disorder detection (κ ≥ 0.88) and for classification in metabolic acidosis by LLM-S (κ = 0.85–0.94) (Table 3).

Accurate classification of metabolic acidosis and associated disorders remains challenging, particularly due to variability in interpreting the Δ/Δ ratio across guidelines [13,15,26], and physiological factors [8,15,27]. The Δ/Δ ratio should be considered as a supportive tool rather than a standalone diagnostic criterion, requiring correlation with clinical and laboratory findings [15,26]. This approach likely contributed to the higher agreement seen with LLM-S, where interpretation followed the same criteria as the conventional method.

The improved performance of the LLM-S (human-in-the-loop) approach, particularly in APSD analysis, underscores the importance of strict user supervision. Such oversight allows results to be nuanced, errors in the application of compensation equations to be identified, inconsistencies in compensatory changes generated by the LLM to be detected, and small numerical deviations to be weighed appropriately. This highlights that LLMs can make (and do make) errors, and that users must validate or reject outputs based on physiological knowledge of acid–base regulation and its disorders. One potential educational use of this tool is to provide medical students or residents with clinical cases to interpret under supervision (LLM-S), and then to repeat the exercise without supervision (LLM-I). This sequential practice could enable users to recognize typical LLM errors (often the same mistakes humans may also commit) while reinforcing the need to apply medical knowledge and illustrating the risks of blind reliance on such tools.

Mixed disorder classification also depends on the choice of compensation equations and the acceptable deviation ranges. In our study, we applied a threshold of ±2 mmol/L for HCO_3_^−^ or ±2 mmHg for PaCO_2_, which may have influenced LLM performance, particularly in the absence of contextual clinical data. Notably, only Copilot made formula-related errors, and these were limited to two cases (1%). In the sensitivity analysis, the proportion of mixed disorders decreased substantially (from 34% to 20.5%); however, a marked change was observed only with ChatGPT, whereas Gemini and Copilot showed performance results like the a priori analysis.

A sub-analysis of severe secondary disorders showed that ChatGPT in LLM-S achieved perfect agreement (κ = 1.0), regardless of whether the disorder was primary or secondary, underscoring the educational value of LLMs. Given that calculation errors and “math anxiety” are known barriers in health care students [28], LLMs may help reduce cognitive load while reinforcing understanding of physiological principles.

Specialized LLMs, such as the ABG-trained ChatGPT model evaluated by Turan et al. (2025) [29], in which two physicians assessed ABG interpretation in critically ill patients, have achieved near-perfect accuracy across multiple parameters using Stewart’s methodology. In that study, the reported accuracy was 0.98 for identifying the primary disorder, 1.0 for compensation status, and 1.0 for anion gap status. Although the methodology differed and direct comparisons cannot be made, our study also demonstrated strong accuracy with the LLM-S approach, consistently achieving values above 0.90 (except for Gemini and Copilot in APSD, where agreement was still ≥0.83). While specialized training may reduce variability related to differences in equations and reference values, we adopted a physiological approach that may be more accessible to students than Stewart’s method [30]. However, general-purpose LLMs remain relevant in education due to their accessibility and capacity to provide human-like explanation. This interpretability offers an advantage over some machine learning models with higher raw accuracy but limited transparency [31].

Although concerns persist regarding LLM reasoning capacity in complex clinical contexts [32,33], our structured prompting approach, with conditional, disorder-specific queries, intends to mitigate inconsistencies and prevent hallucinations [2,4,34,35]. Our primary focus, however, was educational accuracy, aiming to use the LLMs as an interactive learning tool that allows students to work through cases and receive reliable feedback [7,36].

Strengths of this study include several methodological and educational aspects. First, it is among the first investigations to systematically evaluate three general-purpose LLMs (ChatGPT, Copilot, and Gemini) for ABG interpretation, applying a standardized prompting system and a human-in-the-loop approach that clearly improved diagnostic accuracy. Second, the internal validity was reinforced by a formal sample size calculation based on established methods. Third, the design was reproducible and accessible: prompts were simple, literature-based, and formulated in Spanish, making replication feasible in educational contexts across Spanish-speaking countries. Fourth, the inclusion of both pathological and normal ABG cases is a relevant pedagogical element, as it trains students not only to recognize complex disorders but also to correctly identify physiological states. Finally, the analytical rigor was strengthened by the post hoc use of the imbalance-robust *R_k_* coefficient, macro- and micro-averaged diagnostic measures, and sub-analyses in extreme pH and sensitivity analysis, all of which support the robustness of the findings.

However, there are several limitations to this study. One is that we did not evaluate whether LLM-assisted interpretation improves learners understanding of acid–base disorders or, conversely, fosters an overreliance on model support. Addressing this question will require randomized controlled trials comparing the performance of learners using LLM assistance against a control group without assistance. Nevertheless, our findings highlight the potential of this approach, as LLM-assisted evaluation demonstrated expert-level accuracy. We plan to design an educational intervention study to further explore this issue.

Another important limitation was the exclusion of cases with hypoalbuminemia, as well as the predominance of relatively simple disorders in non-critically ill patients. The most challenging scenarios in arterial blood gas interpretation occur in critically ill patients, particularly those with strong ion difference disturbances or alterations in weak non-volatile acids, where the Stewart approach may be more attractive [37]. However, this simplification and the predominance of emergency department cases was deliberate, since our primary goal was to evaluate the diagnostic accuracy of LLM-assisted evaluation when used by medical trainees or physicians with limited prior experience in this field. Caution should be exercised when extrapolating our findings, as there was a time interval between the collection of clinical chemistry data and the arterial blood gas analysis. Although this delay was generally short, it may have introduced discrepancies in rapidly changing physiological states, particularly among unstable patients.

The perfect agreement obtained between the two evaluators also deserves comment. Because the second evaluator was aware of the sampling scheme (40 cases per category), we cannot fully exclude a potential bias in their classification. Nonetheless, strict adherence to the study protocol and the fact that all compensation calculations were performed using software likely contributed to this result. The study protocol also predefined adjudication by a third evaluator in the event of disagreement, although this was ultimately not required. Interestingly, Turan et al. (2025) [29] likewise reported perfect agreement between evaluators under similar conditions. Still, in a more clinically realistic setting (i.e., bedside evaluation), lower agreement would be expected.

A further limitation of the study design was that each student was assigned to a single LLM, which could confound model performance with operator-dependent factors. Despite our efforts to mitigate this risk through prompt standardization and an algorithmic, literature-based approach, future research adopting a crossover design is necessary to isolate true model-level performance. We considered it relevant to evaluate not only ChatGPT but also Gemini and Copilot, as in our country some universities provide access to these models with fewer restrictions compared to free access. In addition, all evaluations were performed with paid subscription plans to ensure stable access to the latest model versions. Free tiers also provide the same core models and only downgrade once usage limits are exceeded; however, because our study involved hundreds of prompts per model, paid access was necessary. In typical educational or clinical use, far fewer prompts would be required, making free access more feasible.

The study was also conducted entirely in Spanish, which may limit generalizability, since subtle language-related differences could persist. However, several published studies [38,39,40], along with an additional non–peer-reviewed report [41], suggest that prompts in Spanish do not systematically impair performance. Another limitation is that we applied equal sampling across the five diagnostic categories, which ensured balanced representation but deviates from real-world prevalence, where some disorders are more common than others. Moreover, the study was conducted at a single tertiary university hospital, which provided consistent protocols and high-quality data but limits external validity, as case mix and clinical practices may differ across institutions. Future studies should adopt prevalence-weighted sampling and multicenter designs to enhance reproducibility and generalizability.

## 5. Conclusions

This study demonstrates that LLMs can achieve strong agreement with expert interpretation in ABG analysis, including accurate recognition of cases without acid–base disorders. Accuracy was consistently higher when LLM calculations were interpreted under supervision (LLM-S) compared with unsupervised interpretation (LLM-I), particularly in the classification of mixed disorders.

Our aim was to develop an accessible and reproducible prompting system that highlights both the computational accuracy and explanatory potential of LLMs. Beyond diagnostic performance, their step-by-step calculations and explanations suggest potential educational benefits, such as reducing arithmetic errors and alleviating “math anxiety” among medical trainees.

Given the single-center, retrospective design and simplified case selection, these findings should be interpreted as preliminary. While they support the promise of LLMs as educational tools, further prospective and controlled studies are needed to establish their broader impact on learning outcomes and eventual clinical applicability.

## Figures and Tables

**Table 2:** Patient demographics and arterial blood gas findings.

	NoABD	MAc	RAc	MAk	RAk
Female	14 (35%)	13 (33%)	18 (45%)	21 (52.5%)	16 (40%)
Age, years	50 (38–64)	45 (36–56)	53 (38–65)	54 (42–69)	47 (41–62)
pH	7.40 (7.38–7.42)	7.30 (7.23–7.33)	7.31 (7.26–7.34)	7.49 (7.47–7.52)	7.48 (7.46–7.51)
PaCO_2_, mmHg	38 (36–40)	28 (21–31)	50 (48–53)	43 (40–46)	25 (22–28)
HCO_3_^−^, mmol/L	24 (23–25)	12 (10–14)	25 (23–28)	33 (30–36)	20 (17–25)
Anion gap, mmol/L	8.9 (7.0–11.0)	18.2 (14.5–21.2)	9.3 (6.9–10.6)	8.0 (5.9–9.4)	10.1 (9.1–11.0)
Isolated primary disorder frequency	NA	18 (45%)	26 (65%)	8 (20%)	34 (85%)

NoABD, no acid–base disorder. MAc, metabolic acidosis; MAk, metabolic alkalosis; RAc, respiratory acidosis; RAk, respiratory alkalosis; NA, not applicable. Age, pH, PaCO_2_, HCO_3_^−^ and anion gap are expressed as median (interquartile range).

**Table 3:** Agreement (Cohen’s κ value) between LLM-assisted and conventional evaluation of acid–base disorders.

		APD (κ CI95%)	APD-a (κ CI95%)	APSD (κ CI95%)	AMA (κ CI95%)
LLM-I	ChatGPT	κ = 0.91 (0.84–0.98), *R*_k_ = 0.91	κ = 0.96 (0.89–1.0), *R*_k_ = 0.96	κ = 0.65 (0.60–0.70), *R*_k_ = 0.67	κ = 0.55 (0.39–0.72), *R*_k_ = 0.58
	Copilot	κ = 0.95 (0.88–1.0), *R*_k_ = 0.95	κ = 0.98 (0.91–1.0), *R*_k_ = 0.98	κ = 0.61 (0.56–0.66), *R*_k_ = 0.63	κ = 0.48 (0.32–0.63), *R*_k_ = 0.52
	Gemini	κ = 0.88 (0.81–0.95), *R*_k_ = 0.89	κ = 0.94 (0.87–1.0), *R*_k_ = 0.94	κ = 0.62 (0.57–0.67), *R*_k_ = 0.63	κ = 0.76 (0.58–0.95), *R*_k_ = 0.77
LLM-S	ChatGPT	κ = 0.89 (0.83–0.96), *R*_k_ = 0.89	κ = 0.92 (0.85–0.99), *R*_k_ = 0.92	κ = 0.91 (0.85–0.96), *R*_k_ = 0.91	κ = 0.85 (0.66–1.0), *R*_k_ = 0.86
	Copilot	κ = 0.92 (0.85–0.99), *R*_k_ = 0.92	κ = 0.94 (0.88–1.0), *R*_k_ = 0.95	κ = 0.81 (0.75–0.86), *R*_k_ = 0.81	κ = 0.86 (0.67–1.0), *R*_k_ = 0.86
	Gemini	κ = 0.92 (0.85–0.99), *R*_k_ = 0.92	κ = 0.97 (0.90–1.0), *R*_k_ = 0.97	κ = 0.81 (0.76–0.86), *R*_k_ = 0.81	κ = 0.94 (0.75–1.0), *R*_k_ = 0.94

APD, agreement on primary disorder; APD-a, agreement on primary disorder with detection; APSD, agreement on both primary and secondary disorders regardless of order; AMA, agreement on the classification of metabolic acidosis. CI95%, confidence interval at 95%; LLM-I, LLM-assisted evaluation with interpretation. LLM-S, LLM-assisted evaluation with supervision. κ and *R*_k_ values rounded to two decimal places.

**Table 4:** Sub-analysis of agreement (Cohen’s κ value) between LLM-assisted and conventional evaluation.

		pH ≤ 7.30 or pH ≥ 7.50		Severe Secondary Disorder	
		APD (κ CI95%)	APSD (κ CI95%)	APD (κ CI95%)	APSD (κ CI95%)
LLM-I	ChatGPT	κ = 0.94 (0.80–1.0), *R*_k_ = 0.94	κ = 0.71 (0.61–0.82), *R*_k_ = 0.72	κ = 0.95 (0.70–1.0), *R*_k_ = 0.95	κ = 0.91 (0.71–1.0), *R*_k_ = 0.92
	Copilot	κ = 0.96 (0.82–1.0), *R*_k_ = 0.96	κ = 0.60 (0.50–0.70), *R*_k_ = 0.61	κ = 0.95 (0.70–1.0), *R*_k_ = 0.95	κ = 0.68 (0.52–0.84), *R*_k_ = 0.73
	Gemini	κ = 0.90 (0.76–1.0), *R*_k_ = 0.91	κ = 0.70 (0.60–0.80), *R*_k_ = 0.71	κ = 0.74 (0.50–0.98), *R*_k_ = 0.74	κ = 0.78 (0.60–0.97), *R*_k_ = 0.80
LLM-S	ChatGPT	κ = 0.96 (0.82–1.0), *R*_k_ = 0.96	κ = 1.0 (0.89–1.0), *R*_k_ = 1.0	κ = 0.89 (0.64–1.0), *R*_k_ = 0.90	κ = 1.0 (0.78–1.0), *R*_k_ = 1.0
	Copilot	κ = 0.96 (0.82–1.0), *R*_k_ = 0.96	κ = 0.84 (0.74–0.94), *R*_k_ = 0.85	κ = 0.89 (0.64–1.0), *R*_k_ = 0.90	κ = 0.74 (0.55–0.93), *R*_k_ = 0.75
	Gemini	κ = 0.96 (0.82–1.0), *R*_k_ = 0.96	κ = 0.91 (0.81–1.0), *R*_k_ = 0.91	κ = 0.90 (0.66–1.0), *R*_k_ = 0.90	κ = 0.75 (0.57–0.93), *R*_k_ = 0.78

APD, agreement on primary disorder; APSD, agreement on primary and secondary disorders irrespective of order; CI95%, confidence interval at 95%; LLM-I, LLM-Assisted evaluation with interpretation. LLM-S, LLM-Assisted evaluation with supervision. κ and *R*_k_ values rounded to two decimal places.

**Table 5:** Macro-averaged performance metrics and accuracy of LLM-assisted evaluation.

			Se.	Sp.	PPV	NPV	Accuracy (CI95%)
LLM-I	ChatGPT	APD	0.93	0.98	0.94	0.98	0.93 (0.89–0.96)
		APD-a	0.97	0.99	0.97	0.99	0.97 (0.93–0.99)
		APSD	0.74	0.96	0.72	0.96	0.69 (0.92–0.75)
		AMA	0.76	0.90	0.61	0.89	0.69 (0.55–0.81)
	Copilot	APD	0.96	0.99	0.97	0.98	0.96 (0.92–0.98)
		APD-a	0.99	1.0	0.99	1.0	0.98 (0.96–1.0)
		APSD	0.70	0.96	0.66	0.96	0.66 (0.58–0.72)
		AMA	0.52	0.88	0.59	0.87	0.63 (0.48–0.76)
	Gemini	APD	0.91	0.98	0.92	0.98	0.91 (0.86–0.94)
		APD-a	0.95	0.99	0.96	0.99	0.95 (0.91–0.98)
		APSD	0.70	0.96	0.67	0.96	0.62 (0.59–0.73)
		AMA	0.86	0.95	0.70	0.94	0.85 (0.72–0.93)
LLM-S	ChatGPT	APD	0.92	0.98	0.93	0.98	0.92 (0.87–0.95)
		APD-a	0.94	0.98	0.94	0.98	0.94 (0.89–0.96)
		APSD	0.95	0.99	0.92	0.99	0.92 (0.87–0.95)
		AMA	0.95	0.97	0.94	0.96	0.91 (0.79–0.97)
	Copilot	APD	0.94	0.98	0.95	0.98	0.94 (0.89–0.96)
		APD-a	0.96	0.99	0.96	0.99	0.96 (0.92–0.98)
		APSD	0.85	0.98	0.79	0.98	0.83 (0.77–0.88)
		AMA	0.95	0.97	0.77	0.96	0.91 (0.79–0.97)
	Gemini	APD	0.94	0.98	0.95	0.98	0.94 (0.89–0.96)
		APD-a	0.98	0.99	0.98	0.99	0.98 (0.94–0.99)
		APSD	0.84	0.98	0.79	0.98	0.84 (0.78–0.88)
		AMA	0.94	0.99	0.83	0.99	0.96 (0.87–0.99)

APD, agreement on primary disorder; APD-a, agreement on primary disorder with detection; APSD, agreement on both primary and secondary disorders regardless of order; AMA, agreement on the classification of metabolic acidosis. LLM-I, LLM-assisted evaluation with interpretation. LLM-S, LLM-assisted evaluation with supervision. Se., sensitivity; Sp., specificity; PPV, positive predictive value; NPV, negative predictive value. Values rounded to two decimal places.

## Data Availability

The data presented in this study are available from the corresponding author upon reasonable request.

## Supplementary Materials

The following supporting information can be downloaded at https://www.mdpi.com/article/10.3390/jcm14186676/s1.

## Abbreviations

The following abbreviations are used in this manuscript:

ABG	Arterial blood gas
AG	Anion gap
AMA	Agreement on the classification of metabolic acidosis
APD	Agreement on primary disorder
APD-a	Agreement on primary disorder with detection
APSD	Agreement on primary and secondary disorders
HCO_3_^−^	Bicarbonate
ICU	Intensive care unit
IQR	Interquartile range
LLM-I	LLM-Assisted evaluation with interpretation
LLM-S	LLM-Assisted evaluation with supervision
LLMs	Large language models
MAc	Metabolic acidosis
MAk	Metabolic alkalosis
NPV	Negative predictive value
NoABD	No acid–base disorder
PaCO_2_	Arterial partial pressure of carbon dioxide
pH	Potential of hydrogen
PPV	Positive predictive value
RAc	Respiratory acidosis
RAk	Respiratory alkalosis
SA	Sensitivity analysis
Se.	Sensitivity
Sp.	Specificity
USD	United States dollars
Δ/Δ	Delta/delta ratio

## References

[1] Alberts I.L., Mercolli L., Pyka T., Prenosil G., Shi K., Rominger A., Afshar-Oromieh A. (2023). Large language models (LLM) and ChatGPT: What will the impact on nuclear medicine be?. Eur. J. Nucl. Med. Mol. Imaging.

[2] Yu E., Chu X., Zhang W., Meng X., Yang Y., Ji X., Wu C. (2025). Large Language Models in Medicine: Applications, Challenges, and Future Directions. Int. J. Med. Sci..

[3] Hager P., Jungmann F., Holland R., Bhagat K., Hubrecht I., Knauer M., Vielhauer J., Makowski M., Braren R., Kaissis G. (2024). Evaluation and mitigation of the limitations of large language models in clinical decision-making. Nat. Med..

[4] Yang H.S., Wang F., Greenblatt M.B., Huang S.X., Zhang Y. (2023). AI Chatbots in Clinical Laboratory Medicine: Foundations and Trends. Clin. Chem..

[5] Vrdoljak J., Boban Z., Vilović M., Kumrić M., Božić J. (2025). A Review of Large Language Models in Medical Education, Clinical Decision Support, and Healthcare Administration. Healthcare.

[6] Doumat G., Daher D., Ghanem N.-N., Khater B. (2022). Knowledge and attitudes of medical students in Lebanon toward artificial intelligence: A national survey study. Front. Artif. Intell..

[7] Ejaz H., McGrath H., Wong B.L., Guise A., Vercauteren T., Shapey J. (2022). Artificial intelligence and medical education: A global mixed-methods study of medical students’ perspectives. Digit. Health.

[8] Berend K., de Vries A.P.J., Gans R.O.B. (2014). Physiological Approach to Assessment of Acid–Base Disturbances. N. Engl. J. Med..

[9] Berend K. (2018). Diagnostic Use of Base Excess in Acid–Base Disorders. N. Engl. J. Med..

[10] Adrogué H.J., Madias N.E. (2010). Secondary Responses to Altered Acid-Base Status. J. Am. Soc. Nephrol..

[11] Donner A., Rotondi M.A. (2010). Sample Size Requirements for Interval Estimation of the Kappa Statistic for Interobserver Agreement Studies with a Binary Outcome and Multiple Raters. Int. J. Biostat..

[12] McHugh M.L. (2012). Interrater reliability: The kappa statistic. Biochem. Med..

[13] Rodríguez-Villar S., Do Vale B.M., Fletcher H.M. (2020). El algoritmo de la gasometría arterial: Propuesta de un enfoque sistemático para el análisis de los trastornos del equilibrio ácido-base. Rev. Esp. Anestesiol. Reanim..

[14] Kaufman D. Interpretation of Arterial Blood Gases (ABGs). https://www.thoracic.org/professionals/clinical-resources/critical-care/clinical-education/abgs.php.

[15] Fenves A.Z., Emmett M. (2021). Approach to Patients with High Anion Gap Metabolic Acidosis: Core Curriculum 2021. Am. J. Kidney Dis..

[16] Emmett M., Palmer B. The Delta Anion Gap/Delta HCO_3_ Ratio in Patients with a High Anion Gap Metabolic Acidosis. https://www.uptodate.com/contents/the-delta-anion-gap-delta-hco3-ratio-in-patients-with-a-high-anion-gap-metabolic-acidosis.

[17] Delgado R., Tibau X.-A. (2019). Why Cohen’s Kappa should be avoided as performance measure in classification. PLoS ONE.

[18] Gorodkin J. (2004). Comparing two K-category assignments by a K-category correlation coefficient. Comput. Biol. Chem..

[19] Kautz T., Eskofier B.M., Pasluosta C.F. (2017). Generic performance measure for multiclass-classifiers. Pattern Recognit..

[20] Brinkman J.E., Sharma S. (2025). Physiology, Metabolic Alkalosis. StatPearls [Internet].

[21] Lee S.Y., Koh E.S., Chung S. (2024). #1772 Comparison of interpretation of acid-base disorder in patients with critical illness: Nephrologist versus ChatGPT. Nephrol. Dial. Transplant..

[22] OpenAI Model Release Notes. https://help.openai.com/en/articles/9624314-model-release-notes?utm_source=chatgpt.com#h_8e49be5daa.

[23] Google Gemini 2.5 Flash. https://cloud.google.com/vertex-ai/generative-ai/docs/models/gemini/2-5-flash.

[24] Spataro J. Bringing the Latest Capabilities to Copilot for Microsoft 365 Customers. https://www.microsoft.com/en-us/microsoft-365/blog/2024/04/02/bringing-the-latest-capabilities-to-copilot-for-microsoft-365-customers/.

[25] Gün M. (2025). AI-Assisted Blood Gas Interpretation: A Comparative Study with an Emergency Physician. Am. J. Emerg. Med..

[26] Rastegar A. (2007). Use of the ΔAG/ΔHCO^3−^ Ratio in the Diagnosis of Mixed Acid-Base Disorders. J. Am. Soc. Nephrol..

[27] Rudkin S.E., Grogan T.R., Treger R.M. (2021). The Δ Anion Gap/Δ Bicarbonate Ratio in Early Lactic Acidosis: Time for Another Delta?. Kidney360.

[28] Khasawneh E., Gosling C., Williams B. (2021). What impact does maths anxiety have on university students?. BMC Psychol..

[29] Turan E.İ., Baydemir A.E., Balıtatlı A.B., Şahin A.S. (2025). Assessing the accuracy of ChatGPT in interpreting blood gas analysis results ChatGPT-4 in blood gas analysis. J. Clin. Anesth..

[30] Honore P., Kishen R., Jacobs R., Joannes-Boyau O., De Waele E., De Regt J., Van Gorp V., Boer W., Spapen H. (2014). Facing acid–base disorders in the third millennium—The Stewart approach revisited. Int. J. Nephrol. Renov. Dis..

[31] Ozdemir H., Sasmaz M.I., Guven R., Avci A. (2025). Interpretation of acid–base metabolism on arterial blood gas samples via machine learning algorithms. Ir. J. Med. Sci..

[32] Urda-Cîmpean A.E., Leucuța D.-C., Drugan C., Duțu A.-G., Călinici T., Drugan T. (2025). Assessing the Accuracy of Diagnostic Capabilities of Large Language Models. Diagnostics.

[33] Geetha S.D., Khan A., Khan A., Kannadath B.S., Vitkovski T. (2024). Evaluation of ChatGPT pathology knowledge using board-style questions. Am. J. Clin. Pathol..

[34] Bélisle-Pipon J.-C. (2024). Why we need to be careful with LLMs in medicine. Front. Med..

[35] Yu S., Lee S.-S., Hwang H. (2024). The ethics of using artificial intelligence in medical research. Kosin Med. J..

[36] Tozsin A., Ucmak H., Soyturk S., Aydin A., Gozen A.S., Fahim M.A., Güven S., Ahmed K. (2024). The Role of Artificial Intelligence in Medical Education: A Systematic Review. Surg. Innov..

[37] Masevicius F.D. (2015). Has Stewart approach improved our ability to diagnose acid-base disorders in critically ill patients?. World J. Crit. Care Med..

[38] Ray M., Kats D.J., Moorkens J., Rai D., Shaar N., Quinones D., Vermeulen A., Mateo C.M., Brewster R.C.L., Khan A. (2025). Evaluating a Large Language Model in Translating Patient Instructions to Spanish Using a Standardized Framework. JAMA Pediatr..

[39] Delaunay J., Cusido J. (2024). Evaluating the Performance of Large Language Models in Predicting Diagnostics for Spanish Clinical Cases in Cardiology. Appl. Sci..

[40] Li Z., Shi Y., Liu Z., Yang F., Payani A., Liu N., Du M. (2024). Language Ranker: A Metric for Quantifying LLM Performance Across High and Low-Resource Languages. Proc. AAAI Conf. Artif. Intell..

[41] Zhang Z., Liu Y., Huang W., Mao J., Wang R., Hu H. (2024). MELA: Multilingual Evaluation of Linguistic Acceptability. arXiv.

