# Supplementary material for: Human-in-the-Loop Performance of LLM-Assisted Arterial Blood Gas Interpretation: A Single-Center Retrospective Study
Source: J Clin Med. 2025 Sep 22;14(18):6676. doi: 10.3390/jcm14186676 (PMC12470526; doi:10.3390/jcm14186676)
Supplement: Supplementary file 1 [file jcm-14-06676-s001.zip › jcm-3861866-supplementary.pdf]

**Supplementary Table S1.**

**Micro and macro-averaged performance metrics in LLM assisted evaluation with interpretation (LLM-I).** NoABD, no acid-base disorder; MAc, metabolic acidosis; MAk, metabolic alkalosis; RAc, respiratory acidosis; RAk, respiratory alkalosis. APD, agreement on primary disorder; APSD, agreement on both primary and secondary disorders regardless of order; PPV, positive predictive value; NPV, negative predictive value; F1, F1 score. Macro-Avg, Macro-average. Values rounded to two decimal places.

| | LLM | Disorder (n) | Sensitivity | Specificity | PPV | NPV | F1 |
|---|---|---|---|---|---|---|---|
| APD | ChatGPT | NoABD (40) | 0.98 | 0.99 | 0.98 | 0.99 | 0.98 |
| | | MAc (40) | 1.0 | 0.94 | 0.80 | 1.0 | 0.89 |
| | | RAc (40) | 0.88 | 0.99 | 0.95 | 0.97 | 0.91 |
| | | MAk (40) | 0.98 | 0.99 | 0.98 | 0.99 | 0.98 |
| | | RAk (40) | 0.82 | 1.0 | 1.0 | 0.96 | 0.90 |
| | | Macro-Avg | 0.93 | 0.98 | 0.94 | 0.98 | 0.93 |
| | Copilot | NoABD (40) | 1.0 | 0.99 | 0.98 | 0.99 | 1.0 |
| | | MAc (40) | 1.0 | 0.94 | 0.80 | 1.0 | 0.92 |
| | | RAc (40) | 0.88 | 0.99 | 0.95 | 0.97 | 0.93 |
| | | MAk (40) | 1.0 | 0.99 | 0.98 | 0.99 | 0.99 |
| | | RAk (40) | 0.92 | 1.0 | 1.0 | 0.96 | 0.96 |
| | | Macro-Avg | 0.96 | 0.99 | 0.97 | 0.99 | 0.96 |
| | Gemini | NoABD (40) | 0.90 | 0.99 | 0.97 | 0.98 | 0.94 |
| | | MAc (40) | 0.95 | 0.91 | 0.73 | 0.99 | 0.83 |
| | | RAc (40) | 0.72 | 1.0 | 1.0 | 0.94 | 0.84 |
| | | MAk (40) | 1.0 | 0.99 | 0.95 | 1.0 | 0.98 |
| | | RAk (40) | 0.95 | 0.99 | 0.95 | 0.99 | 0.95 |
| | | Macro-Avg | 0.91 | 0.98 | 0.92 | 0.98 | 0.91 |
| APSD | ChatGPT | NoAbd (40) | 0.98 | 0.99 | 0.98 | 0.99 | 0.98 |
| | | MAc (18) | 0.89 | 0.97 | 0.76 | 0.99 | 0.82 |
| | | RAc (26) | 0.19 | 1.0 | 1.0 | 0.89 | 0.32 |
| | | MAk (8) | 0.75 | 0.98 | 0.60 | 0.99 | 0.67 |
| | | RAk (34) | 0.12 | 1.0 | 1.0 | 0.85 | 0.21 |
| | | MAc/RAc (19) | 0.95 | 0.93 | 0.60 | 0.99 | 0.73 |
| | | MAc/RAk (16) | 0.94 | 0.91 | 0.47 | 0.99 | 0.62 |
| | | RAc/MAk (6) | 1.0 | 0.94 | 0.33 | 1.0 | 0.50 |
| | | MAk/RAk (33) | 0.88 | 0.93 | 0.73 | 0.98 | 0.79 |
| | | Macro-Avg | 0.74 | 0.96 | 0.72 | 0.96 | 0.63 |
| | Copilot | NoAbd (40) | 1.0 | 1.0 | 1.0 | 1.0 | 1.0 |
| | | MAc (18) | 1.0 | 0.95 | 0.67 | 1.0 | 0.80 |
| | | RAc (26) | 0.31 | 0.99 | 0.80 | 0.91 | 0.44 |
| | | MAk (8) | 0.75 | 0.92 | 0.29 | 0.99 | 0.41 |
| | | RAk (34) | 0.44 | 1.0 | 1.0 | 0.90 | 0.61 |
| | | MAc/RAc (19) | 0.84 | 0.96 | 0.67 | 0.98 | 0.74 |
| | | MAc/RAk (16) | 0.62 | 0.90 | 0.34 | 0.96 | 0.44 |
| | | RAc/MAk (6) | 1.0 | 0.93 | 0.32 | 1.0 | 0.48 |
| | | MAk/RAk (33) | 0.36 | 0.99 | 0.86 | 0.89 | 0.51 |
| | | Macro-Avg | 0.70 | 0.96 | 0.66 | 0.96 | 0.61 |
| | Gemini | NoAbd (40) | 0.90 | 0.99 | 0.97 | 0.98 | 0.94 |
| | | MAc (18) | 0.94 | 0.95 | 0.63 | 0.99 | 0.76 |
| | | RAc (26) | 0.38 | 0.99 | 0.91 | 0.92 | 0.54 |
| | | MAk (8) | 0.50 | 0.91 | 0.18 | 0.98 | 0.27 |
| | | RAk (34) | 0.47 | 0.99 | 0.89 | 0.90 | 0.62 |
| | | MAc/RAc (19) | 0.84 | 0.98 | 0.84 | 0.98 | 0.84 |
| | | MAc/RAk (16) | 0.81 | 0.90 | 0.41 | 0.98 | 0.54 |
| | | RAc/MAk (6) | 1.0 | 0.94 | 0.35 | 1.0 | 0.52 |
| | | MAk/RAk (33) | 0.42 | 0.98 | 0.82 | 0.90 | 0.56 |
| | | Macro-Avg | 0.70 | 0.96 | 0.67 | 0.96 | 0.62 |

**Supplementary Table S2.**

**Micro and macro-averaged performance metrics in LLM assisted evaluation with supervision (LLM-S)**. NoABD, no acid-base disorder; MAc, metabolic acidosis; MAk, metabolic alkalosis; RAc, respiratory acidosis; RAk, respiratory alkalosis. APD, agreement on primary disorder; APSD, agreement on both primary and secondary disorders regardless of order; PPV, positive predictive value; NPV, negative predictive value; F1, F1 score. Macro-Avg, Macro-average. Values rounded to two decimal places.

| | LLM | Disorder (n) | Sensitivity | Specificity | PPV | NPV | F1 |
|---|---|---|---|---|---|---|---|
| APD | ChatGPT | NoABD (40) | 0.80 | 1.0 | 1.0 | 0.95 | 0.89 |
| | | MAc (40) | 1.0 | 0.94 | 0.80 | 1.0 | 0.89 |
| | | RAc (40) | 0.80 | 0.99 | 0.97 | 0.95 | 0.88 |
| | | MAk (40) | 1.0 | 0.99 | 0.98 | 1.0 | 0.99 |
| | | RAk (40) | 0.98 | 0.97 | 0.89 | 0.99 | 0.93 |
| | | Macro-Avg | 0.92 | 0.98 | 0.93 | 0.98 | 0.91 |
| | Copilot | NoABD (40) | 0.92 | 0.99 | 0.97 | 0.98 | 0.95 |
| | | MAc (40) | 1.0 | 0.93 | 0.78 | 1.0 | 0.88 |
| | | RAc (40) | 0.80 | 1.0 | 1.0 | 0.95 | 0.89 |
| | | MAk (40) | 1.0 | 0.99 | 0.98 | 1.0 | 0.99 |
| | | RAk (40) | 0.95 | 1.0 | 1.0 | 0.99 | 0.97 |
| | | Macro-Avg | 0.94 | 0.98 | 0.95 | 0.98 | 0.94 |
| | Gemini | NoABD (40) | 1.0 | 0.99 | 0.98 | 1.0 | 0.99 |
| | | MAc (40) | 0.98 | 0.93 | 0.78 | 0.99 | 0.87 |
| | | RAc (40) | 0.78 | 1.0 | 1.0 | 0.95 | 0.87 |
| | | MAk (40) | 1.0 | 1.0 | 1.0 | 1.0 | 1.0 |
| | | RAk (40) | 0.92 | 0.99 | 0.97 | 0.98 | 0.95 |
| | | Macro-Avg | 0.94 | 0.98 | 0.95 | 0.98 | 0.94 |
| APSD | ChatGPT | NoAbd (40) | 0.80 | 1.0 | 1.0 | 0.95 | 0.89 |
| | | MAc (18) | 1.0 | 0.97 | 0.78 | 1.0 | 0.88 |
| | | RAc (26) | 0.81 | 0.99 | 0.95 | 0.97 | 0.88 |
| | | MAk (8) | 1.0 | 0.99 | 0.89 | 1.0 | 0.94 |
| | | RAk (34) | 0.94 | 0.97 | 0.86 | 0.99 | 0.90 |
| | | MAc/RAc (19) | 1.0 | 1.0 | 1.0 | 1.0 | 1.0 |
| | | MAc/RAk (16) | 1.0 | 0.98 | 0.84 | 1.0 | 0.91 |
| | | RAc/MAk (6) | 1.0 | 1.0 | 1.0 | 1.0 | 1.0 |
| | | MAk/RAk (33) | 0.97 | 0.99 | 0.97 | 0.99 | 0.97 |
| | | Macro-Avg | 0.95 | 0.99 | 0.92 | 0.99 | 0.93 |
| | Copilot | NoAbd (40) | 0.92 | 0.99 | 0.97 | 0.98 | 0.95 |
| | | MAc (18) | 1.0 | 0.94 | 0.62 | 1.0 | 0.77 |
| | | RAc (26) | 0.77 | 1.0 | 1.0 | 0.97 | 0.87 |
| | | MAk (8) | 1.0 | 0.96 | 0.50 | 1.0 | 0.67 |
| | | RAk (34) | 0.85 | 1.0 | 1.0 | 0.97 | 0.92 |
| | | MAc/RAc (19) | 0.95 | 0.99 | 0.95 | 0.99 | 0.95 |
| | | MAc/RAk (16) | 0.75 | 0.96 | 0.63 | 0.98 | 0.69 |
| | | RAc/MAk (6) | 0.83 | 0.97 | 0.50 | 0.99 | 0.62 |
| | | MAk/RAk (33) | 0.58 | 0.99 | 0.95 | 0.92 | 0.72 |
| | | Macro-Avg | 0.85 | 0.98 | 0.79 | 0.98 | 0.79 |
| | Gemini | NoAbd (40) | 1.0 | 0.99 | 0.98 | 1.0 | 0.99 |
| | | MAc (18) | 1.0 | 0.98 | 0.82 | 1.0 | 0.90 |
| | | RAc (26) | 0.69 | 0.99 | 0.95 | 0.96 | 0.80 |
| | | MAk (8) | 0.62 | 0.93 | 0.28 | 0.98 | 0.38 |
| | | RAk (34) | 0.88 | 1.0 | 1.0 | 0.98 | 0.94 |
| | | MAc/RAc (19) | 1.0 | 0.99 | 0.90 | 1.0 | 0.65 |
| | | MAc/RAk (16) | 1.0 | 0.98 | 0.84 | 1.0 | 0.91 |
| | | RAc/MAk (6) | 0.83 | 0.96 | 0.38 | 0.99 | 0.53 |
| | | MAk/RAk (33) | 0.48 | 0.99 | 0.94 | 0.91 | 0.64 |
| | | Macro-Avg | 0.84 | 0.98 | 0.79 | 0.98 | 0.78 |

**Supplementary Table S3.**

**Sensitivity Analysis.** A sensitivity analysis was performed by modifying the tolerances in the compensation formulas. The tolerance for Winters equation remained unchanged at ±2. For the remaining formulas (metabolic alkalosis, respiratory acidosis, and respiratory alkalosis), the tolerance was modified to ±5. This analysis, referred to as SA (sensitivity analysis), was applied to APSD and AMA using the LLM-I method with SA as the reference standard. APD and APD-a were omitted, as the classification of primary disorders is not affected by changes in tolerance thresholds.

APSD, agreement on both primary and secondary disorders regardless of order; AMA, agreement on the classification of metabolic acidosis. CI95%, confidence interval at 95%. Values rounded to two decimal places.

| | **LLM** | **APSD (κ CI95%)** | **AMA (κ CI95%)** |
|---|---|---|---|
| LLM-I | ChatGPT | κ= 0.51 (0.47-0.56), $R_k$= 0.54 | κ= 0.49 (0.32-0.66), $R_k$= 0.52 |
| | Copilot | κ= 0.59 (0.55-0.64), $R_k$= 0.61 | κ= 0.42 (0.27-0.58), $R_k$= 0.46 |
| | Gemini | κ= 0.63 (0.58-0.68), $R_k$= 0.65 | κ= 0.72 (0.53-0.91), $R_k$= 0.73 |

**Supplementary Table S4.**

**Spanish-language prompts used in LLM-assisted evaluation.** LLM-I, LLM-assisted evaluation with interpretation; LLM-S, LLM-assisted evaluation with supervision.

| Prompt | LLM-I | LLM-S |
|---|---|---|
| P1 | Identifica si existe o no existe un trastorno ácido base considerando los siguientes resultados (los valores de referencia se encuentran entre paréntesis): pH= XX (7.35-7.45), $PaCO_2$= XX mmHg (35-45 mmHg), $HCO_3^-$= XX mmol/L (22-26 mmol/L). El resultado de los electrolitos séricos es: sodio= XX mmol/L, cloro= XX mmol/L. En caso de identificar un trastorno ácido base, determina cual es el trastorno primario. | |
| P2 | Realiza el cálculo de la compensación de PaCO2 en la acidosis metabólica y determina si existe un trastorno respiratorio agregado. | Realiza el cálculo de la compensación de $PaCO_2$ en la acidosis metabólica. |
| P3 | Determina e interpreta el valor de la brecha aniónica. | Determina el valor de la brecha aniónica. |
| P4 | Determina e interpreta la relación delta/delta utilizando los datos del valor delta brecha aniónica y delta $HCO_3^-$. | Determina la relación delta/delta utilizando los datos del valor delta brecha aniónica y delta $HCO_3^-$. |
| P5 | Realiza el cálculo de la compensación de $HCO_3^-$ en la acidosis respiratoria **aguda/crónica** y determina si existe un trastorno metabólico agregado. | Realiza el cálculo de la compensación de HCO3 en la acidosis respiratoria **aguda/crónica.** |
| P6 | Realiza el cálculo de la compensación de $PaCO_2$ en la alcalosis metabólica y determina si existe un trastorno respiratorio agregado. | Realiza el cálculo de la compensación de $PaCO_2$ en la alcalosis metabólica. |
| P7 | Realiza el cálculo de la compensación de $HCO_3^-$ en la alcalosis respiratoria **aguda/crónica** y determina si existe un trastorno metabólico agregado. | Realiza el cálculo de la compensación de $HCO_3^-$ en la alcalosis respiratoria **aguda/crónica.** |

**Supplementary Table S5.**

**LLM versions used in each workflow.** LLM-I, LLM-assisted evaluation with interpretation; LLM-S, LLM-assisted evaluation with supervision. Gemini-2.5-flash became generally available on June 17, 2025; therefore, most LLM-S evaluations were conducted with Gemini-1.5-flash.

| LLM | Evaluation method | Dates of use | Version employed |
|---|---|---|---|
| ChatGPT | LLM-I | Jan 22 – Feb 28, 2025 | GPT-4o (OpenAI) |
| ChatGPT | LLM-S | Jun 3 – Jun 20, 2025 | GPT-4o (OpenAI) |
| Copilot | LLM-I | Jan 22 – Feb 28, 2025 | GPT-4 (Microsoft, exact release not public) |
| Copilot | LLM-S | Apr 2 – Jun 20, 2025 | GPT-4 (Microsoft, exact release not public) |
| Gemini | LLM-I | Jan 22 – Feb 28, 2025 | Gemini-1.5-flash (Google) |
| Gemini | LLM-S | Apr 2 – Jun 20, 2025 | Gemini-1.5-flash (Google), Gemini-2.5-flash (Google, released Jun 17) |

**Supplementary Figure S1.**

**Agreement (Cohen's κ value) on both primary and secondary disorders regardless of order between a priori análisis (APA) and sensitivity análisis (SA).** NoABD, no acid-base disorder; MAc, metabolic acidosis; MAk, metabolic alkalosis; RAc, respiratory acidosis; RAk, respiratory alkalosis. Cells indicate agreement frequencies: green = concordant cases, orange = discordant cases.

**SA, κ=0.81**

| SA \ APA | NoABD | MAc | RAc | MAk | RAk | MAc/RAc | MAc/RAk | RAc/MAk | MAk/RAk |
|---|---|---|---|---|---|---|---|---|---|
| NoABD | 40 | 0 | 0 | 0 | 0 | 0 | 0 | 0 | 0 |
| MAc | 0 | 18 | 0 | 0 | 0 | 0 | 0 | 0 | 0 |
| RAc | 0 | 0 | 26 | 0 | 0 | 6 | 0 | 0 | 0 |
| MAk | 0 | 0 | 0 | 8 | 0 | 0 | 0 | 2 | 20 |
| RAk | 0 | 0 | 0 | 0 | 34 | 0 | 1 | 0 | 4 |
| MAc/RAc | 0 | 0 | 0 | 0 | 0 | 13 | 0 | 0 | 0 |
| MAc/RAk | 0 | 0 | 0 | 0 | 0 | 0 | 15 | 0 | 0 |
| RAc/MAk | 0 | 0 | 0 | 0 | 0 | 0 | 0 | 4 | 0 |
| MAk/RAk | 0 | 0 | 0 | 0 | 0 | 0 | 0 | 0 | 9 |
| Total=200 | 40 | 18 | 26 | 8 | 34 | 19 | 16 | 6 | 33 |

**Supplementary Figure S2.**

**Agreement (Cohen's κ value) on primary disorder between LLM-assisted with interpretation (LLM-I) and conventional method (APD)**. (a) ChatGPT; (b) Copilot; (c) Gemini. Conv, conventional evaluation (column titles), LLM, large language model assisted evaluation (row titles); NoABD, no acid-base disorder; MAc, metabolic acidosis; MAk, metabolic alkalosis; RAc, respiratory acidosis; RAk, respiratory alkalosis. Cells indicate agreement frequencies: green = concordant cases, orange = discordant cases.

**(a) ChatGPT, κ=0.91**

| LLM \ Conv | NoABD | MAc | RAc | MAk | RAk |
|---|---|---|---|---|---|
| NoABD | 39 | 0 | 1 | 0 | 0 |
| MAc | 0 | 40 | 4 | 0 | 6 |
| RAc | 1 | 0 | 35 | 1 | 0 |
| MAk | 0 | 0 | 0 | 39 | 1 |
| RAk | 0 | 0 | 0 | 0 | 33 |
| Total=200 | 40 | 40 | 40 | 40 | 40 |

**(b) Copilot, κ=0.95**

| LLM \ Conv | NoABD | MAc | RAc | MAk | RAk |
|---|---|---|---|---|---|
| NoABD | 40 | 0 | 0 | 0 | 0 |
| MAc | 0 | 40 | 4 | 0 | 3 |
| RAc | 0 | 0 | 35 | 0 | 0 |
| MAk | 0 | 0 | 1 | 40 | 0 |
| RAk | 0 | 0 | 0 | 0 | 37 |
| Total=200 | 40 | 40 | 40 | 40 | 40 |

**(c) Gemini, κ=0.88**

| LLM \ Conv | NoABD | MAc | RAc | MAk | RAk |
|---|---|---|---|---|---|
| NoABD | 36 | 0 | 1 | 0 | 0 |
| MAc | 2 | 38 | 10 | 0 | 2 |
| RAc | 0 | 0 | 29 | 0 | 0 |
| MAk | 2 | 0 | 0 | 40 | 0 |
| RAk | 0 | 2 | 0 | 0 | 38 |
| Total=200 | 40 | 40 | 40 | 40 | 40 |

**Supplementary Figure S3.**

**Agreement (Cohen's κ value) on primary disorder between LLM-assisted with interpretation (LLM-I) and conventional method even if it appeared as a secondary disorder (APD-a).** (a) ChatGPT; (b) Copilot; (c) Gemini. Conv, conventional evaluation (column titles), LLM, large language model assisted evaluation (row titles). NoABD, no acid-base disorder. MAc, metabolic acidosis; MAk, metabolic alkalosis; RAc, respiratory acidosis; RAk, respiratory alkalosis. Cells indicate agreement frequencies: green = concordant cases, orange = discordant cases.

**(a) ChatGPT, κ=0.96**

| LLM \ Conv | NoABD | MAc | RAc | MAk | RAk |
|---|---|---|---|---|---|
| NoABD | 39 | 0 | 1 | 0 | 0 |
| MAc | 0 | 40 | 3 | 0 | 1 |
| RAc | 1 | 0 | 36 | 0 | 0 |
| MAk | 0 | 0 | 0 | 40 | 1 |
| RAk | 0 | 0 | 0 | 0 | 38 |
| Total=200 | 40 | 40 | 40 | 40 | 40 |

**(b) Copilot, κ=0.98**

| LLM \ Conv | NoABD | MAc | RAc | MAk | RAk |
|---|---|---|---|---|---|
| NoABD | 40 | 0 | 0 | 0 | 0 |
| MAc | 0 | 40 | 2 | 0 | 1 |
| RAc | 0 | 0 | 38 | 0 | 0 |
| MAk | 0 | 0 | 0 | 40 | 0 |
| RAk | 0 | 0 | 0 | 0 | 39 |
| Total=200 | 40 | 40 | 40 | 40 | 40 |

**(c) Gemini, κ=0.94**

| LLM \ Conv | NoABD | MAc | RAc | MAk | RAk |
|---|---|---|---|---|---|
| NoABD | 36 | 0 | 1 | 0 | 0 |
| MAc | 2 | 40 | 5 | 0 | 0 |
| RAc | 0 | 0 | 34 | 0 | 0 |
| MAk | 2 | 0 | 0 | 40 | 0 |
| RAk | 0 | 0 | 0 | 0 | 40 |
| Total=200 | 40 | 40 | 40 | 40 | 40 |

**Supplementary Figure S4.**

**Agreement (Cohen's κ value) on both primary and secondary disorders regardless of order between LLM-assisted with interpretation (LLM-I) and conventional method (APSD).** (a) ChatGPT; (b) Copilot; (c) Gemini. Conv, conventional evaluation (column titles), LLM, large language model assisted evaluation (row titles); NoABD, no acid-base disorder; MAc, metabolic acidosis; MAk, metabolic alkalosis; RAc, respiratory acidosis; RAk, respiratory alkalosis. Cells indicate agreement frequencies: green = concordant cases, orange = discordant cases.

**(a) ChatGPT, κ=0.65**

| LLM \ Conv | NoABD | MAc | RAc | MAk | RAk | MAc/RAc | MAc/RAk | RAc/MAk | MAk/RAk |
|---|---|---|---|---|---|---|---|---|---|
| NoABD | 39 | 0 | 1 | 0 | 0 | 0 | 0 | 0 | 0 |
| MAc | 0 | 16 | 3 | 0 | 1 | 0 | 1 | 0 | 0 |
| RAc | 0 | 0 | 5 | 0 | 0 | 0 | 0 | 0 | 0 |
| MAk | 0 | 0 | 0 | 6 | 0 | 0 | 0 | 0 | 4 |
| RAk | 0 | 0 | 0 | 0 | 4 | 0 | 0 | 0 | 0 |
| MAc/RAc | 1 | 2 | 9 | 0 | 0 | 18 | 0 | 0 | 0 |
| MAc/RAk | 0 | 0 | 0 | 0 | 17 | 0 | 15 | 0 | 0 |
| RAc/MAk | 0 | 0 | 8 | 2 | 1 | 1 | 0 | 6 | 0 |
| MAk/RAk | 0 | 0 | 0 | 0 | 11 | 0 | 0 | 0 | 29 |
| Total=200 | 40 | 18 | 26 | 8 | 34 | 19 | 16 | 6 | 33 |

**(b) Copilot, κ=0.61**

| LLM \ Conv | NoABD | MAc | RAc | MAk | RAk | MAc/RAc | MAc/RAk | RAc/MAk | MAk/RAk |
|---|---|---|---|---|---|---|---|---|---|
| NoABD | 40 | 0 | 0 | 0 | 0 | 0 | 0 | 0 | 0 |
| MAc | 0 | 18 | 2 | 0 | 1 | 0 | 6 | 0 | 0 |
| RAc | 0 | 0 | 8 | 0 | 0 | 2 | 0 | 0 | 0 |
| MAk | 0 | 0 | 0 | 6 | 0 | 0 | 0 | 0 | 15 |
| RAk | 0 | 0 | 0 | 0 | 15 | 0 | 0 | 0 | 0 |
| MAc/RAc | 0 | 0 | 8 | 0 | 0 | 16 | 0 | 0 | 0 |
| MAc/RAk | 0 | 0 | 0 | 0 | 16 | 1 | 10 | 0 | 2 |
| RAc/MAk | 0 | 0 | 8 | 2 | 0 | 0 | 0 | 6 | 4 |
| MAk/RAk | 0 | 0 | 0 | 0 | 2 | 0 | 0 | 0 | 12 |
| Total=200 | 40 | 18 | 26 | 8 | 34 | 19 | 16 | 6 | 33 |

**(c) Gemini, κ=0.62**

| LLM \ Conv | NoABD | MAc | RAc | MAk | RAk | MAc/RAc | MAc/RAk | RAc/MAk | MAk/RAk |
|---|---|---|---|---|---|---|---|---|---|
| NoABD | 36 | 0 | 1 | 0 | 0 | 0 | 0 | 0 | 0 |
| MAc | 1 | 17 | 5 | 0 | 0 | 1 | 3 | 0 | 0 |
| RAc | 0 | 0 | 10 | 0 | 0 | 1 | 0 | 0 | 0 |
| MAk | 1 | 0 | 0 | 4 | 0 | 0 | 0 | 0 | 17 |
| RAk | 0 | 0 | 0 | 0 | 16 | 0 | 0 | 0 | 2 |
| MAc/RAc | 0 | 0 | 3 | 0 | 0 | 16 | 0 | 0 | 0 |
| MAc/RAk | 1 | 1 | 0 | 0 | 16 | 1 | 13 | 0 | 0 |
| RAc/MAk | 0 | 0 | 7 | 4 | 0 | 0 | 0 | 6 | 0 |
| MAk/RAk | 1 | 0 | 0 | 0 | 2 | 0 | 0 | 0 | 14 |
| Total=200 | 40 | 18 | 26 | 8 | 34 | 19 | 16 | 6 | 33 |
